# Photocatalytic Reduction of Cr(VI) by MOF@Azo-COF Under Strongly Acidic Conditions

**DOI:** 10.3390/ijms27188174

**Published:** 2026-09-14

**Authors:** Xiaolong Gong, Guojian Ren, Qi Zhou, Linglong Yi, Qingxiang Wu, Yingjie Hua, Qinhe Pan

**Affiliations:** 1 Key Laboratory of Advanced Materials of Tropical Island Resources, Ministry of Education, School of Chemistry and Chemical Engineering, Hainan University, Haikou 570228, China; 25110805000102@hainanu.edu.cn (X.G.); 25110805000044@hainanu.edu.cn (Q.Z.); 22220951350036@hainanu.edu.cn (L.Y.); 24210817000034@hainanu.edu.cn (Q.W.); 2 School of Materials Science and Engineering, Hainan University, Haikou 570228, China; 3 State Key Laboratory of Inorganic Synthesis and Preparative Chemistry, College of Chemistry, Jilin University, Changchun 130012, China; 4 Key Laboratory of Electrochemical Energy Storage and Energy Conversion of Hainan Province, School of Chemistry and Chemical Engineering, Hainan Normal University, Haikou 571158, China; 070018@hainnu.edu.cn; 5 National Key Laboratory of Uranium Resources Exploration-Mining and Nuclear Remote Sensing, East China University of Technology, Nanchang 330013, China

**Keywords:** strongly acidic environment, photocatalytic reduction of Cr(VI), azo–covalent–organic framework, metal–organic framework

## Abstract

Cr(VI) poses a serious threat to the ecosystem and human health; in order to efficiently remove Cr(VI), a stable MOF@COF core-shell photocatalyst (MOF-808@TpAzo) was developed. Through a post-modification strategy, MOF-808 was functionalized with amino groups on its surface. Subsequently, the TpAzo-based covalent organic framework (COF) was grown in-situ on its surface, successfully yielding the core-shell structured MOF-808@TpAzo composite. Under highly acidic conditions, the MOF-808@TpAzo composite shows an excellent photocatalytic reduction of Cr(VI) performance, which is significantly better than that of its single component. Especially, as little as 5 mg of the catalyst, 99.42% of Cr(VI), was removed from the acidic simulated wastewater within 120 min, and the material exhibited excellent cycling stability. The highly effective catalysis is attributed to the heterojunction formed via the covalent connection between the MOF and COF components, promoting charge separation and transport. This work provides a new alternative for developing stable photocatalysts capable of the reduction of Cr(VI) in strongly acidic environments.

## 1. Introduction

The rapid development of the industry has led to severe environmental pollution problems [1], in which heavy metal pollution [2] poses a serious threat to the ecosystem [3] and human health [4]. Chromium (Cr), a heavy metal widely used in industrial sectors including electroplating [5], printing [6] and dyeing, leather [7], and steel manufacturing [8], is particularly prone to pollution [9]. Chromium mainly exists in aquatic environments in two oxidation states: Cr(VI) [10] and Cr(III) [11]. Cr(VI) has emerged as one of the most hazardous heavy metal contaminants in surface water due to its strong oxidizing property [12], high mobility [13], and difficult degradability [14]. It is well-established that Cr(III) exhibits a significantly lower toxicity and environmental persistence compared with Cr(VI) [15]. Hence, the exploration of the highly efficient catalysis of Cr(VI) toward Cr(III) keeps being a significant challenge [16]. Solar photocatalysis offers an efficient route for solar-to-chemical energy conversion [17]. As an emerging technology, photocatalytic reduction represents a promising approach under mild conditions [18], which has advantages such as a low energy consumption [19], relatively low cost [20], and no risk of secondary pollution [21], and is an effective way to treat chromium-containing wastewater [22].

In order to achieve the highly efficient photocatalytic reduction of Cr(VI), several critical factors must be addressed, including the recombination of photogenerated electron pairs [23] and holes [24], as well as the limited visible light utilization [25]. Beside the key factors, the catalytic environment should also be considered, because the actual industrial wastewater is usually strongly acidic [26]. Although a strongly acidic environment can provide more favorable thermodynamic conditions for the photocatalytic reduction of Cr(VI), such harsh conditions compromise the structural stability of photocatalysts [27]. As the familiar catalysis, metal oxides including TiO_2_ [28] and ZnO [29] suffer from the problem of acidic instability. For the metal sulfide (CdS [30] and ZnS [31]), they are hindered because of the biotoxicity [32] and stability [33]. Therefore, in strongly acidic environments, the development of photocatalysts that possess both high stability and efficient Cr(VI) reduction performance simultaneously keeps being challenging [34].

Covalent–organic frameworks (COFs) [35] feature highly ordered conjugated structure, endowing it with an excellent visible light absorption capacity [36] and photogenerated electron transport efficiency [37]. Furthermore, through the rational design of organic monomers [38], their electronic structures can be precise, making them promising candidates for photocatalysis. For instance, two sp^2^ carbon-conjugated COF, namely, HDU-107 and HDU-108, performed exceptionally well in the photocatalytic reduction of Cr(VI) [39]. Nonetheless, when employed as a photocatalyst, a single COF component often frequently suffers from a low separation efficiency between the photogenerated charges and holes [40,41]. In order to solve the above-mentioned problem, the adoption of composite materials could be the alternative approach. As a representative example of composite materials, by combining TiO_2_ with COF to form an S-shaped heterojunction, Zhang’s research group achieved the photocatalytic synthesis of H_2_O_2_ and furoic acid [42]. Jerry J. Wu et al. fabricated a COF and CdS heterojunction by using a simple sonochemical method to achieve the photocatalytic degradation of bisphenol A [43]. Although integrating COFs with traditional semiconductors can broaden the application scope of COF itself and enhance its photocatalytic performance, there are still some deficiencies in the composite of other materials with COFs. The conventional physical mixing approach leads to insufficiently tight interface contact [44], and reduced electronic transmission efficiency [45], and, even when COFs are combined with inorganic semiconductor materials, it still fails to overcome the inherent shortcomings of inorganic semiconductor materials. For instance, after TiO_2_ is combined, it still only responds to ultraviolet light [46] and has a relatively weak stability under extreme conditions; similarly, as for CdS, after the combination, it still cannot change its own photo-corrosion [47] and toxicity issues [48]. Therefore, the development of a suitable component that can combine with COF to enhance photocatalytic activity remains a critical issue.

Metal–organic frameworks (MOFs) [49], as one kind of porous crystal materials, have attracted considerable attention in the field of photocatalysis due to their high specific surface area [50], controllable pore structure [51], and light absorption capacity [52]. To enhance the photocatalytic performance, the combination between MOFs and COFs and the construction of heterojunctions can be a feasible solution [53]. Compared with physical mixing, the core-shell structure through the chemical bond combination of MOFs and COFs facilitates the charge transfer rate between the two components, enhancing the photocatalytic performance [54]. The reported results showed that MOF@COF core-shell materials mainly rely on the Schiff base reaction that the −NH_2_ group present on the organic ligand of the MOFs and react with aldehyde-functionalized COFs [55]. This reaction provides the efficient method for the combination of MOFs and COFs. For example, Zhang Fengming’s research group proposed a post-modification approach for the synthesis MOF-808@TpPa-1-COF composites, which showed the high hydrogen evolution rate under visible light [56]. In addition, besides keeping the structural stability under strongly acidic conditions, how to improve the catalytic capacity through the functional introduction remains challenging [57].

**Scheme 1:**
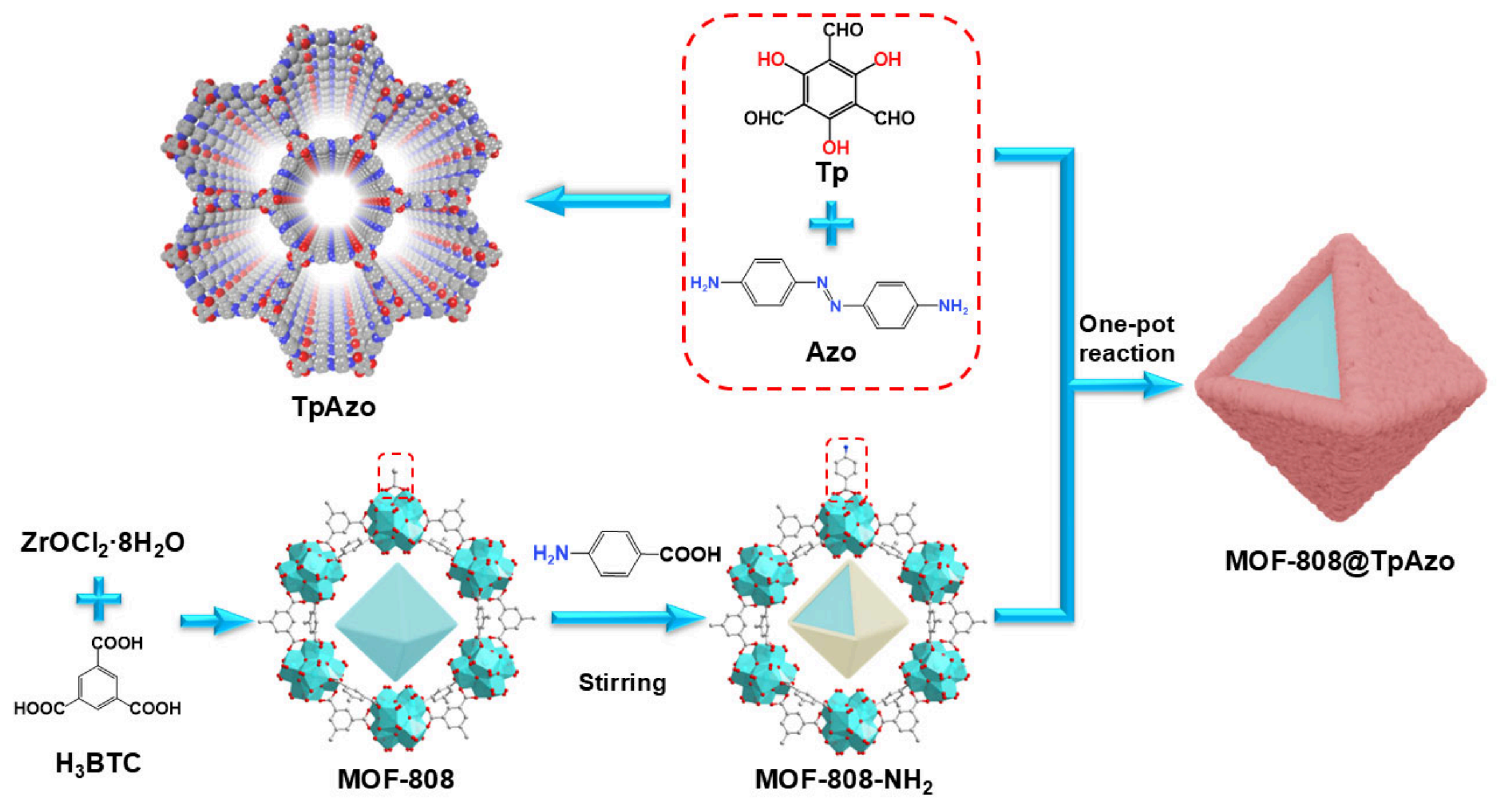
The synthesis of MOF-808@TpAzo schematic diagram.

In this work, benefiting from the wide light absorption range [58], narrow band gap structure [59], and excellent photoelectron transfer ability [60], the Azo-COF (named as TpAzo) is introduced to be combined with MOF-808 for the construction of MOF-808@TpAzo with a core-shell structure (Scheme 1). The composite material shows excellent stability in strongly acidic solutions and various organic solvents. The catalytic results show that the MOF-808@TpAzo composite achieves a 99.42% removal of 50 ppm Cr(VI) in pH = 0 strongly acidic simulated industrial wastewater within 120 min, representing 2.7 times and 1.6 times that of single MOF-808 or TpAzo, respectively. This work provides a new approach for the development of highly efficient Cr(VI) photocatalytic materials that are suitable for strongly acidic environments.

## 2. Results

### 2.1. The Characterizations of MOF-808@TpAzo

**Figure 1:**
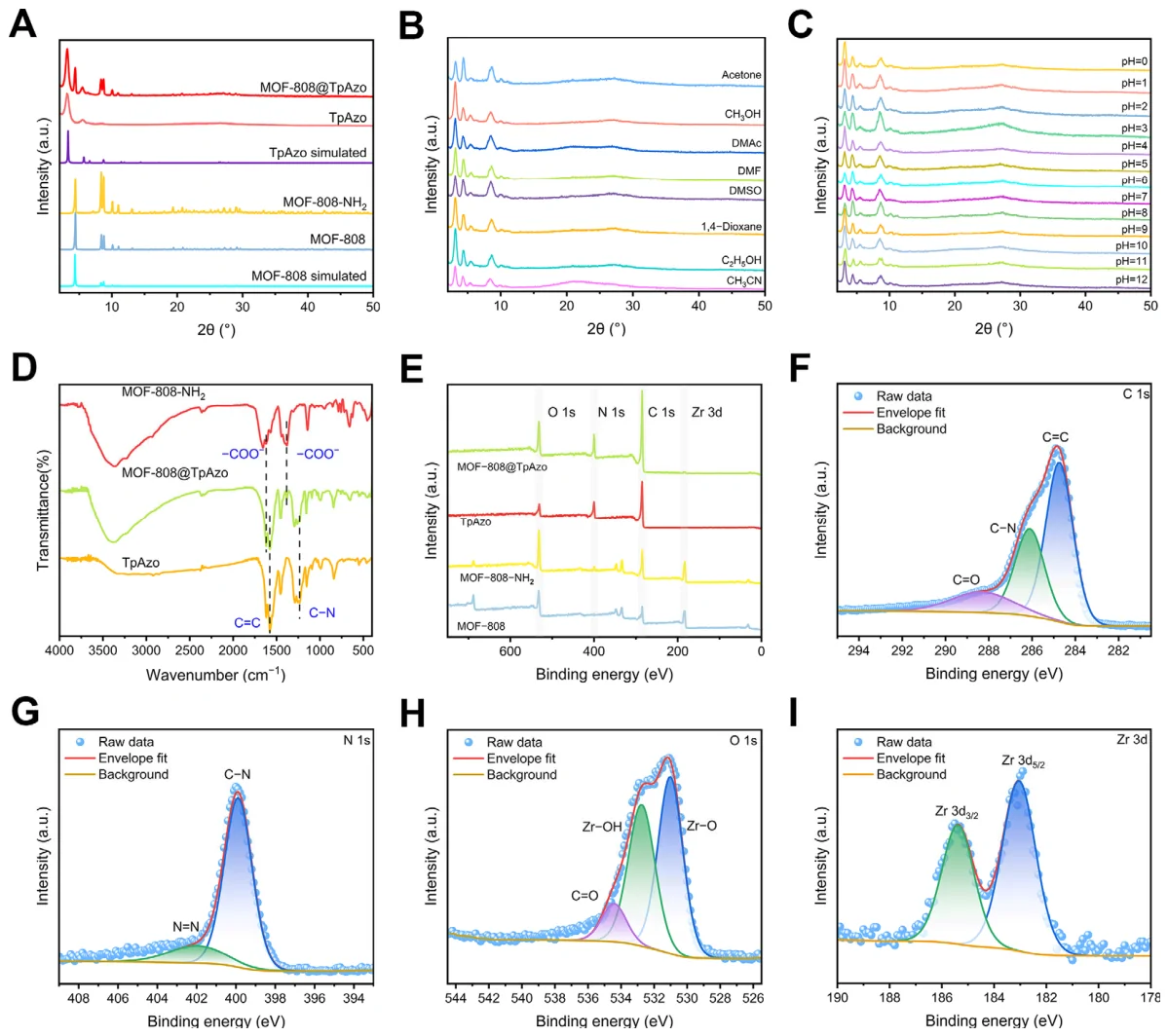
(**A**) The powder X-ray diffraction patterns of MOF-808@TpAzo, TpAzo, MOF-808-NH_2_, and MOF-808, the simulated data of TpAzo and MOF-808 were derived from the reported results [61,62]. (**B**) For different organic solvents and (**C**) for pH conditions, the X-ray diffraction patterns of MOF-808@TpAzo after 24 h immersion are presented. (**D**) The FT-IR spectra of MOF-808, TpAzo, and MOF-808@TpAzo. (**E**) The XPS of MOF-808, MOF-808-NH_2_, TpAzo, and MOF-808@TpAzo. High-resolution deconvoluted spectra of C 1s (**F**), N 1s (**G**), O 1s (**H**), and Zr 3d (**I**).

In order to characterize the crystal structures of the MOF-808, amino-MOF-808 (MOF-808-NH_2_), TpAzo, and MOF-808@TpAzo composites, the powder X-ray diffraction (PXRD) measurement was performed. As shown in Figure 1A, the characteristic diffraction peaks of MOF-808 are highly consistent with the PXRD pattern derived from the crystal structure simulation, confirming the phase purity of as-synthesized MOF-808. After modification with para-aminobenzoic acid, the PXRD pattern of MOF-808-NH_2_ matches well with that of simulated data, which demonstrates that the introduction of the −NH_2_ group preserves the structural integrity of MOF. Referring to the MOF-808@TpAzo composite, the PXRD pattern shows distinct characteristic diffraction peaks at 3.3° and 5.7° of TpAzo, and the characteristic diffraction peaks of MOF-808 can also be maintained, confirming the successful synthesis of the MOF-808@TpAzo composite.

To evaluate the structural stability of the composites, MOF-808@TpAzo were immersed in aqueous solutions with pH 0–12 respectively (adjusted with HCl or NaOH), and various organic solvents for 24 h. As shown in Figure 1B,C, the PXRD patterns of the soaked samples indicated that MOF-808 and TpAzo still maintain their structure in the MOF-808@TpAzo composite, which confirmed that the composite material had excellent structural stability and its robustness in a complex reaction environment.

By comparing the FT-IR spectra of the MOF-808, TpAzo, and MOF-808@TpAzo composites (Figure 1D), the stretching vibration peaks at 1619 cm^−1^ and 1384 cm^−1^ in MOF-808-NH_2_ are assigned to the −COO^−^ existing in MOF-808, the stretching vibration peak at 1233 cm^−1^ in TpAzo arises from the vibration of the C−N bond, and the strong peak at 1578 cm^−1^ formed by the stretching of the C=C bond is due to the formation of *β*-ketoenamine between Tp and Azo. In addition, the MOF-808-NH_2_ FT-IR spectra of the amino group was found in the −NH_2_ characteristic peak, confirming the successful amination of MOF-808 (Figure S1). In the MOF-808@TpAzo composite material, it can be observed that the tensile vibration peaks at 1378 cm^−1^ and 1624 cm^−1^ are assigned to −COO^−^ in MOF-808-NH_2_, and the tensile vibration peaks at 1575 cm^−1^ and 991 cm^−1^ correspond to C=C and C−N in TpAzo, respectively. The FT-IR data proved that the MOF-808@TpAzo composite material has been synthesized.

In order to analyze the elemental composition, X-ray photoelectron spectroscopy (XPS) of MOF-808, TpAzo, and MOF-808@TpAzo was performed (Figure 1E). The complete XPS spectrum of MOF-808@TpAzo indicates the presence of C, N, O, and Zr elements. The Zr 3d peak at 183.0 eV of MOF-808 is relatively high, while the Zr 3d peak of MOF-808@TpAzo is not obvious. This may be attributed to the thicker outer TpAzo shell, which attenuates the Zr 3d peaks [63]. This can also indirectly prove the formation of the core-shell structure. The three peaks 284.7 eV, 286.1 eV, and 288.4 eV in the C 1s spectrum of MOF-808@TpAzo are due to C=C, C−N, and C=O (Figure 1F). In the N 1s XPS spectrum, two peaks appear at 399.9 eV and 402.0 eV, corresponding to the C−N of *β*-ketoenamine at the TpAzo junction and the N=N in the Azo group, respectively (Figure 1G). In the O 1s spectrum of MOF-808@TpAzo, 531 eV, 532.8 eV, and 534.4 eV belong to the C=O, Zr−OH, and Zr−O bonds, respectively (Figure 1H). Two peaks, Zr 3d_3/2_ at 185.38 eV and Zr 3d_5/2_ at 183.04 eV, can be observed in the Zr 3d spectrum, indicating the presence of Zr in MOF-808@TpAzo (Figure 1I). The XPS analysis results indicated that all characteristic peaks of MOF-808 compared to TpAzo were displayed in the composite material, further confirming the successful construction of the MOF-808@TpAzo core-shell structure.

**Figure 2:**
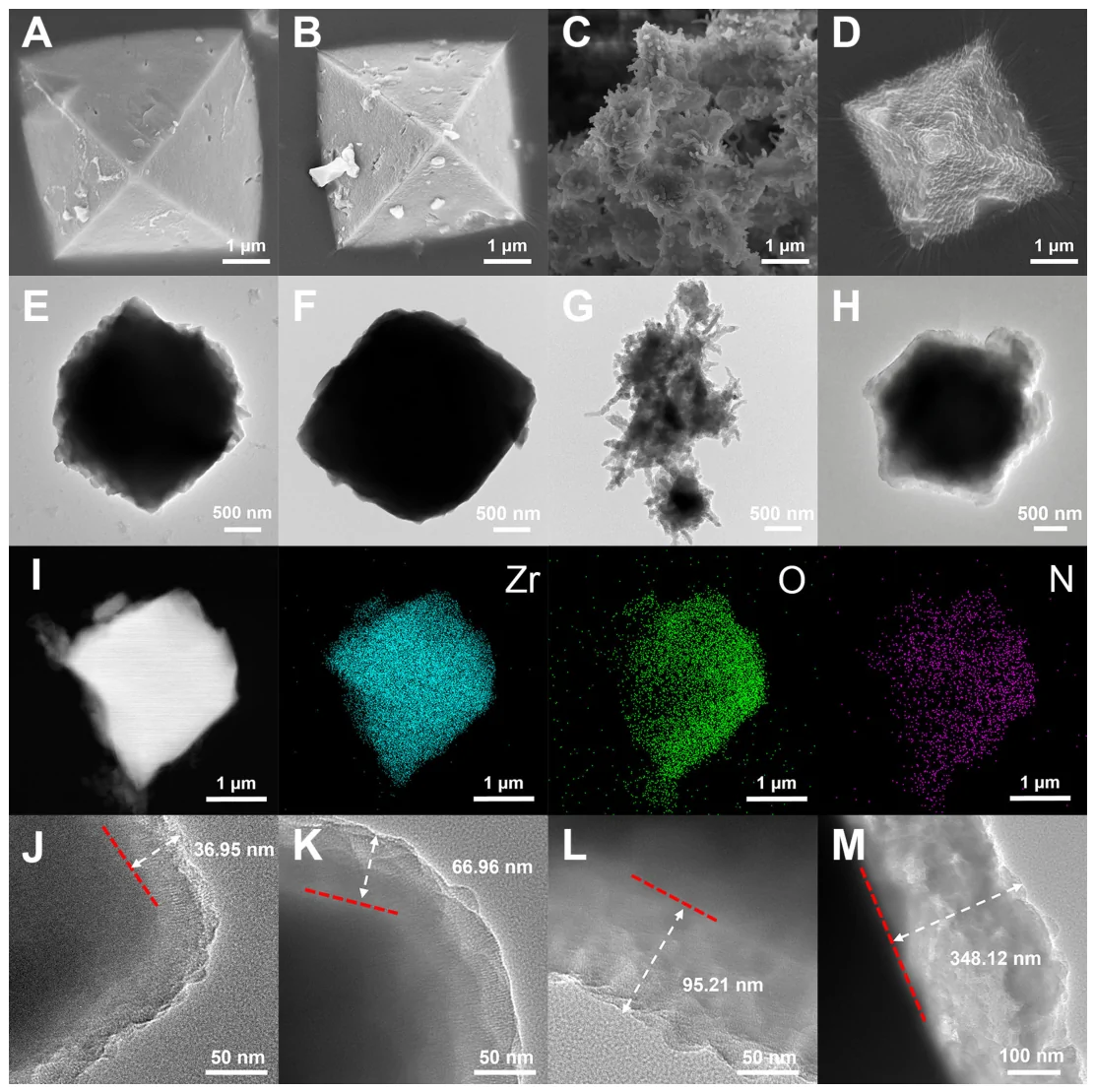
(**A**–**D**) MOF-808, MOF-808-NH_2_, TpAzo, and MOF-808@TpAzo SEM images. (**E**–**H**) MOF-808, MOF-808-NH_2_, TpAzo, and MOF-808@TpAzo FETEM images. (**I**) FETEM image of MOF-808@TpAzo and corresponding elemental mapping images. (**J**–**M**) FETEM images of MOF-808@TpAzo composites prepared at MOF-808 to TpAzo ratios of 2:1 (**J**), 1:1 (**K**), 1:2 (**L**), and 1:4 (**M**). Red dotted line: Interface between MOF-808 and TpAzo; White arrow: Distance from the interface between MOF-808 and TpAzo to the surface of MOF-808@TpAzo.

The morphologies of MOF-808, MOF-808-NH_2_, TpAzo, and MOF-808@TpAzo were observed by using scanning electron microscopy (SEM). As shown in Figure 2A–D, MOF-808 exhibited a regular octahedral structure with a smooth surface. After modification with 4-aminobenzoic acid, MOF-808-NH_2_ still retained the octahedral morphology, as shown in Figure 2B. Furthermore, by comparing the FETEM elemental mapping images of MOF-808 and MOF-808-NH_2_, it can also be demonstrated that the modification strategy after aminoation is feasible (Figures S2 and S3). This indicates that the amination process preserves the original framework structure of MOF-808. In contrast, TpAzo manifested as an irregular flake-like morphology. The FETEM image of TpAzo displayed distinct lattice fringes (Figure S4), which verified that TpAzo possesses high crystallinity. This result is consistent with the characteristic sharp diffraction peak near approximately 3° in the PXRD pattern. The SEM image of the MOF-808@TpAzo composite demonstrated that the material retained the octahedral shape. However, the surface was no longer smooth and was transformed into a state covered with an irregular flake-like morphology. This morphological alteration successfully confirmed the in-situ growth of TpAzo on the surface of the aminated MOF-808, thereby forming a core-shell structure. Furthermore, elemental mapping analysis clearly indicated that elements C, N, O, and Zr were uniformly distributed on the MOF-808@TpAzo composite.

The microstructures of MOF-808, MOF-808-NH_2_, TpAzo, and the optimized MOF-808@TpAzo composite were further characterized by field emission transmission electron microscopy (FETEM). As shown in Figure 2E, F, MOF-808 and MOF-808-NH_2_ all exhibit well-defined octahedral crystals with smooth surfaces, confirming that the amino functionalization does not cause any damage to the framework structure. In contrast, TpAzo (Figure 2G) shows an irregular flake-like morphology, and its high-resolution FETEM image reveals distinct lattice fringes, which is consistent with its strong diffraction peak at ~3° in the PXRD pattern and corroborates its high crystallinity (Figure S4). For the MOF-808@TpAzo heterostructure (Figure 2H), a distinct core-shell structure was observed: the dark octahedral core corresponds to MOF-808, whereas the lighter outer layer is a uniformly coated TpAzo shell layer. At the same time, there is a close and seamless interface between the core and the shell, indicating a strong covalent binding ability between them. This evidence clearly indicates that TpAzo successfully achieved in-situ growth on the amino-activated MOF-808, forming a clearly structured core-shell composite material, in which both components maintained their crystal structures. Further, the elemental distribution of MOF-808@TpAzo was investigated through the energy-dispersive spectroscopy (EDS) of FETEM (Figure 2I). The EDS images showed that Zr from the MOF core was mainly concentrated in the central octahedral region, while N from the TpAzo shell was mainly distributed in the outer layer, and O was evenly distributed throughout the structure, providing strong evidence for the core-shell structure. These results indicate that TpAzo was uniformly grown on MOF-808 rather than physically mixed. Combined with FETEM and PXRD analyses, the EDS results further confirmed the successful construction of the covalently bonded MOF-808@TpAzo heterogeneous structure.

Furthermore, a series of MOF-808@TpAzo samples were prepared by adjusting the mass ratio of MOF-808 to TpAzo (e.g., 2:1, 1:1, 1:2, and 1:4). The PXRD pattern indicates that the relative intensities of the characteristic diffraction peaks of the two components change regularly with the variation in the mass ratio (Figure S5). When the ratio of MOF-808 to TpAzo ranges between 2:1, 1:1, and 1:2, the characteristic peak of MOF-808 remained well-preserved, whereas the intensity of the TpAzo peak increased accordingly. When the ratio increased from 1:2 to 1:4, the excessive thickness of the COF layer led to the complete encapsulation of MOF-808, and the intensity of the characteristic peak of MOF-808 weakened significantly. It is noteworthy that, at a mass ratio of MOF-808 to TpAzo of 1:2, the characteristic diffraction peaks of both components in the composite material are clearly visible and of an appropriate intensity, indicating that its structural integrity could be the best. Therefore, the composite samples with the optimized ratio of MOF-808:TpAzo = 1:2 were selected for subsequent measurement.

Through the FETEM images of MOF-808@TpAzo with different ratios of MOF-808-NH_2_ to TpAzo usage (Figure 2J–M), it can be observed that TpAzo with good crystallinity grows on the outer layer of MOF-808, and the thickness of the TpAzo layer gradually increases with the increase in COF usage. When the MOF and COF package dosage proportions are 2:1, 1:1, 1:2, and 1:4, the corresponding outer TpAzo layer thicknesses of MOF-808@TpAzo is 36.95 nm, 66.96 nm, 95.21 nm, and 348.12 nm, respectively. As shown in the PXRD patterns, the intensities of the characteristic diffraction peaks of TpAzo increase in a series of MOF-808@TpAzo composites, which is consistent with the increase in TpAzo thickness. This result indicates TpAzo successfully grows on the surface of MOF-808, yielding a covalently bonded MOF@COF core-shell structure.

### 2.2. Energy Band Structure Analysis

**Figure 3:**
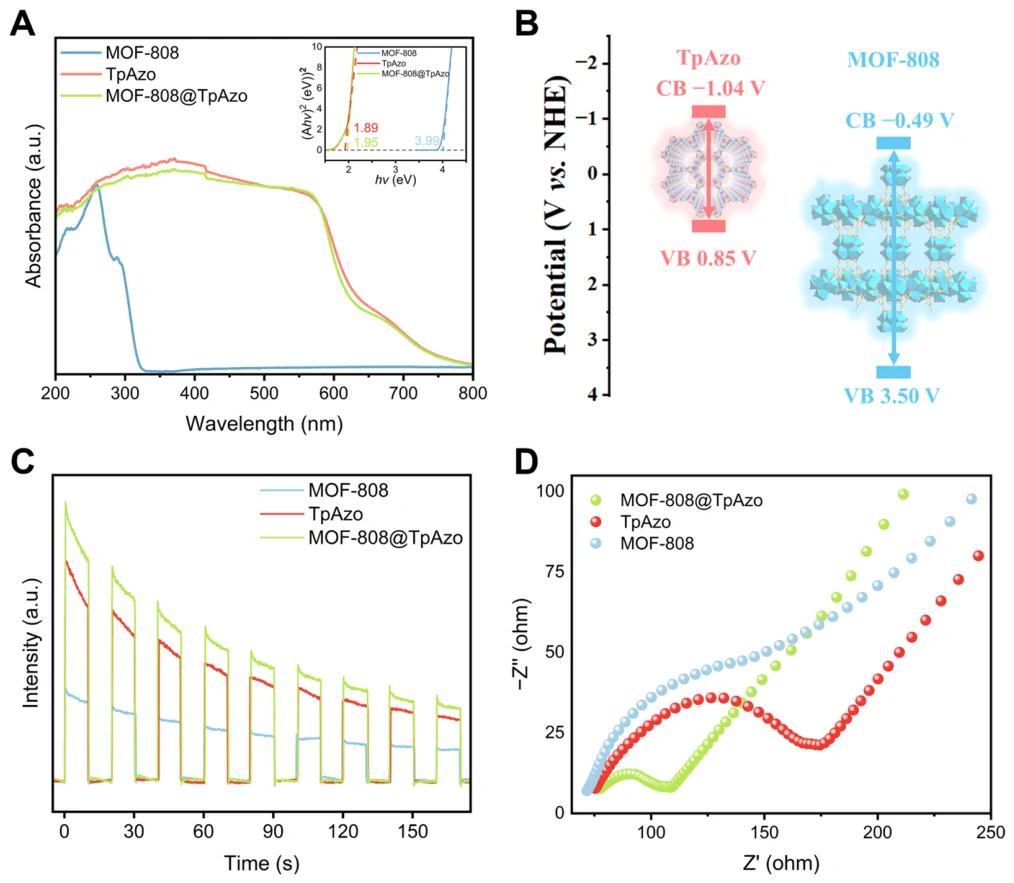
(**A**) UV-Vis DRS and corresponding Tauc plots of MOF-808, TpAzo, and MOF-808@TpAzo. (**B**) Energy band diagrams corresponding to MOF-808 and TpAzo. (**C**) Photocurrent responses. (**D**) EIS spectra of MOF-808, TpAzo, and MOF-808@TpAzo.

The photoelectric properties and band relationship of the MOF-808, TpAzo, and MOF-808@TpAzo composites were analyzed by ultraviolet-visible absorption spectroscopy coupled with Mott-Schottky testing. Through UV-Vis diffuse reflectance spectroscopy tests, MOF-808@TpAzo shows a distinct red color, retains the optical properties of TpAzo, and demonstrates a good light absorption capacity in the visible region (Figure 3A). The Tauc plot shows that the bandgaps of MOF-808, TpAzo, and MOF-808@TpAzo are 3.99 eV, 1.89 eV, and 1.95 eV. In the M-S diagram, both MOF-808 and TpAzo show positive slopes, proving that both MOF-808 and TpAzo are N-type semiconductors (Figure S6). The EFB values of MOF-808 and TpAzo, referenced to saturated calomel electrodes, were −0.59 V and −1.14 V, respectively. In N-type semiconductors, the potential of the conduction band (CB) is 0.1 V less than the EFB value of the semiconductor [64]. Therefore, the corresponding ECB values of MOF-808 and TpAzo versus the saturated calomel electrode are −0.49 V and −1.04 V, respectively. The band diagrams of MOF-808 and TpAzo are shown in the Figure 3B, indicating that MOF-808@TpAzo forms a typical type II heterojunction and the band structure can meet the requirements of photocatalysis. This indicates that the combination of the two materials facilitates the separation of photogenerated electron-hole pairs, thereby enhancing the photocatalytic performance.

### 2.3. Photoelectrochemical Performance Analysis

A series of photoelectric performance tests were conducted to explore the potential photocatalytic property of MOF-808@TpAzo. Transient photocurrent tests demonstrated that, as compared with MOF-808 and TpAzo, MOF-808@TpAzo exhibited a stronger photocurrent intensity during several consecutive light on/off cycle responses, which demonstrates a better photogenerated charge transfer efficiency in the composite material (Figure 3C). Electrochemical impedance spectroscopy (EIS) shows that MOF-808@TpAzo has a smaller interface charge transfer resistance than either MOF-808 or TpAzo (Figure 3D). The photoelectrochemical test results indicated that the MOF-808@TpAzo composites could effectively improve the separation and transport of photogenerated charge-holes, thereby enhancing the photocatalytic efficiency.

### 2.4. Photocatalytic Reduction of Cr(VI) Under Different Conditions

**Figure 4:**
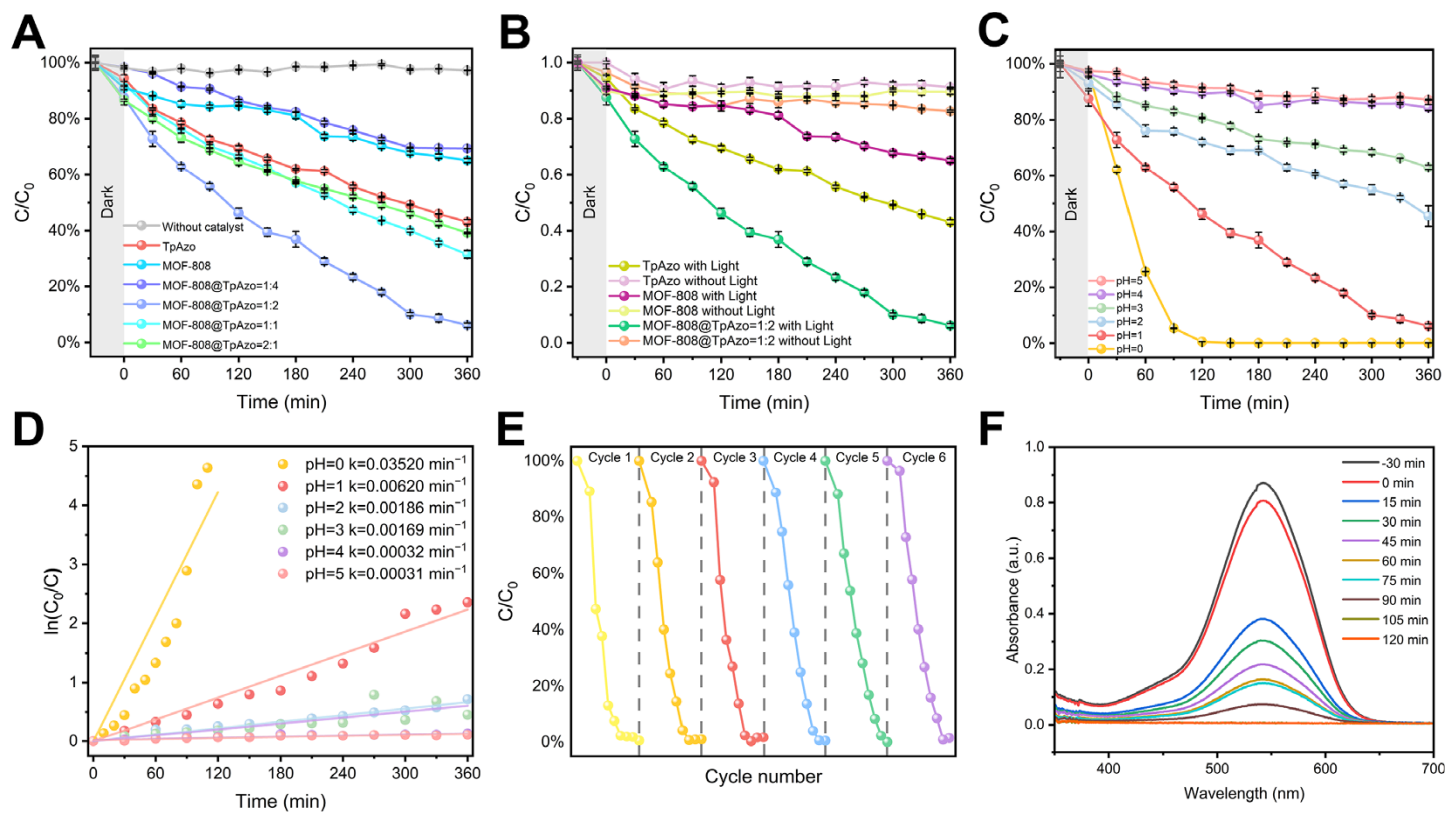
Photocatalytic reduction of Cr(VI) at different (**A**) materials, (**B**) lighting conditions, (**C**) initial pH, and (**D**) reaction constants corresponding to different pH conditions. (**E**) Photocatalytic cycle. (**F**) Ultraviolet absorption spectra of simulated wastewater samples under photocatalytic reduction at different times.

The photocatalytic performance of MOF-808, TpAzo, and MOF-808@TpAzo under xenon lamp irradiation was investigated (Figure 4A). Without adding any substances as catalysts, the concentration of Cr(VI) remains basically unchanged from the initial concentration. Under the same conditions, the MOF-808@TpAzo composite material has a better ability to remove Cr(VI) than the individual components. For the MOF-808 and TpAzo individually, the removal rates were 34.85% and 56.88%, respectively, while the removal rate of Cr(VI) by MOF-808@TpAzo reached 93.82%. The removal rate of Cr(VI) by MOF-808@TpAzo composite material was 2.7 times and 1.6 times that of MOF-808 (34.85%) and TpAzo (56.88%), respectively. To verify whether the removal of Cr(VI) was achieved through photocatalytic reduction or adsorption, the removal performance of Cr(VI) under light conditions was determined (Figure 4B). The experimental results show that, under light irradiation, the Cr(VI) removal capacity of the same sample is much stronger than that under the condition of avoiding light. As the control experiment, under the condition of avoiding light, the content of Cr(VI) removed by adsorption is relatively low. This result indicates that the main removal of Cr(VI) is attributed to the photocatalysis to reduce Cr(VI) to Cr(III). The Cr 2p XPS spectra of the MOF-808@TpAzo sample after adsorption and light exposure were further determined (Figure S7) [65]. In the Cr 2p spectrum of the MOF-808@TpAzo sample after adsorption, a distinct Cr(VI) peak attributable to Cr(VI) is observed at 578.7 eV, while, in the Cr 2p spectrum of the MOF-808@TpAzo sample after photocatalysis, the characteristic peak of Cr(III) at 576.7 eV is dominant. The intensity of the characteristic peak of Cr(VI) decreased significantly, indicating that the MOF-808@TpAzo sample mainly removed Cr(VI) from the reaction system by a photocatalytic reduction to Cr(III).

The photocatalytic removal ability of Cr(VI) by MOF-808@TpAzo composites synthesized with different dosages of MOF-808 and TpAzo was simultaneously determined. As shown in Figure 4A, the removal rates of Cr(VI) by MOF-808@TpAzo synthesized with the dosage ratios of MOF-808 and TpAzo at 2:1, 1:1, 1:2, and 1:4 were 60.77%, 68.5%, 93.82%, and 30.58%, respectively. The highest removal efficiency was presented when the dosages of MOF-808 and TpAzo were 1:2. This finding is consistent with the inference that the composite material with a ratio of 1:2 could have better performance, as confirmed by PXRD and TEM. When the dosage of MOF-808 and TpAzo gradually increased from 2:1 to 1:2, the Cr(VI) removal capacity of the composite material gradually increased from 60.77% to 93.82%. The reason may be that, as the outer layer of TpAzo gradually increased, the electron transport between TpAzo and MOF-808 enhanced. However, when the outer layer thickness continues to increase, such as the ratio of 1:4, the external light source cannot reach the interface between MOF-808 and TpAzo due to the shielding of the thick TpAzo layer, resulting in a decrease in photocatalytic efficiency.

High concentrations of H^+^ not only participate in the reduction of Cr(VI) as reactants, but also suppress the recombination of photogenerated carriers through the protonation effect [66]. To further investigate the photocatalytic removal of Cr(VI) by MOF-808@TpAzo composite materials at different pH, and determine the optimal pH for photocatalytic reduction, the Cr(VI) efficiency was evaluated across a pH range of 0–5 (Figure 4C). With the continuous increase in pH value, the photocatalytic removal effect of Cr(VI) by the MOF-808@TpAzo composite material gradually decreases. When pH = 0, a 99.42% removal of Cr(VI) can be achieved within 120 min. As the pH gradually increases, the removal rate of Cr(VI) becomes lower until pH = 5; 12.81% Cr(VI) can be removed in 360 min. The corresponding reaction rate constant also decreased from 0.03520 min^−1^ to 0.00031 min^−1^ (Figure 4D). According to Figure S8, Cr(VI) mainly exists as ${\mathrm{HCrO}}_{4}^{-}$ across the tested acidic pH range [67]. The reduction of these species heavily relies on the participation of H^+^, as shown in Formula (1). A higher concentration of H^+^ facilitates the photocatalytic reduction process. When the pH increases, Cr(VI) will be reduced to Cr(OH)_3_ (Formula (2)) and precipitates to attach to the MOF-808@TpAzo composite material, reducing the mass transfer rate and covering the photocatalytic active sites, resulting in a decrease in photocatalytic efficiency. Meanwhile, the Nernst equation was used for thermodynamic analysis. Based on the dominant species ${\mathrm{HCrO}}_{4}^{-}$, the corresponding Nernst equation is shown in Formula (3) (derivation provided in Section S2 Supplementary Formula). The pH-dependent Cr(VI) speciation was further supported by UV-Vis measurements using K_2_Cr_2_O_7_ and K_2_CrO_4_ as independent Cr(VI) sources (Figure S9). At pH 0–3, the absorption maximum gradually shifted from ~250 to 257 nm, consistent with the increasing predominance of ${\mathrm{HCrO}}_{4}^{-}$, while relatively stable spectra were observed at pH 3–5. At pH ≥ 6, the absorption red-shifted, consistent with the transition from ${\mathrm{HCrO}}_{4}^{-}$ to $\mathrm{Cr}O_{4}^{2-}$. These observations agree well with the calculated Cr(VI) speciation and with previous literature reports that Cr(VI) speciation is strongly pH-dependent, with ${\mathrm{HCrO}}_{4}^{-}$ predominating under acidic conditions and $\mathrm{Cr}O_{4}^{2-}$ becoming dominant under alkaline conditions [68]. Thus, the combined spectroscopic, thermodynamic, and literature evidence confirms the strong pH dependence of Cr(VI) speciation. As the concentration of H^+^ increases, the reaction quotient becomes smaller, which makes the reduction potential E(${\mathrm{HCrO}}_{4}^{-}$/Cr^3+^) more positive. This indicates that ${\mathrm{HCrO}}_{4}^{-}$ is thermodynamically more easily reduced to Cr^3+^, which is perfectly consistent with the experimental results. The stability of the photocatalyst under strongly acidic conditions is another key factor for the photocatalytic process. It can be found that the MOF-808@TpAzo composite material can remain stable under strong acidic conditions, which has been proven by PXRD in Figure 1C, ensuring that the catalyst can undergo photocatalytic reduction under conditions that are more conducive to the reduction of Cr(VI) to Cr(III).

(1) $${\mathrm{HCrO}}_{4}^{-}+7H^{+}+3e^{-}\text{→}{\mathrm{Cr}}^{3+}+4H_{2}O$$

(2) $$\mathrm{Cr}O_{4}^{2-}+4H_{2}O+3e^{-}\text{→}\mathrm{Cr}{\left(\mathrm{OH}\right)}_{3}\text{↓}+5{\mathrm{OH}}^{-}$$

(3) $$E={\;E}^{\theta }\;-\;\frac{0.05916}{3}\log\frac{c({\mathrm{Cr}}^{3+})}{c({\mathrm{HCrO}}_{4}^{-})\text{·}{[c(H^{+})]}^{7}}$$

The concentration of Cr(VI) in actual industrial wastewater varies significantly, ranging from ten to several thousand ppm. Exploring the photocatalytic removal efficiency at different initial Cr(VI) concentrations is essential for evaluating the practical applicability and effective concentration range of MOF-808@TpAzo with different degrees of pollution. The photocatalytic removal efficiency of MOF-808@TpAzo at initial Cr(VI) concentrations of 25 ppm, 50 ppm, 75 ppm, and 100 ppm was tested (Figure S10). When the initial Cr(VI) concentrations were 25 ppm and 50 ppm, MOF-808@TpAzo achieved nearly complete removal within 120 min, and the quasi-first-order reaction constants were 0.05257 min^−1^ and 0.05395 min^−1^, respectively. When the initial Cr(VI) concentration was increased to 75 ppm and 100 ppm, after 120 min of light exposure, Cr(VI) still existed in the photocatalytic system, and the removal rates of Cr(VI) decreased to 96.4% and 71.6%, respectively. The quasi-first-order reaction constants decreased to 0.02122 min^−1^ and 0.00869 min^−1^, respectively. The possible reasons, apart from the increase in concentration exceeding the photocatalytic removal capacity limit of MOF-808@TpAzo for Cr(VI), could also be that the increase in Cr(VI) concentration darkens the solution color, hindering the absorption of light by MOF-808@TpAzo. Moreover, increasing the concentration of Cr(VI) leads to blocking the pore and catalytic active site in MOF-808@TpAzo, resulting in a reduced photocatalytic ability.

### 2.5. Reusability and Simulated Wastewater Applicability of MOF-808@TpAzo

To evaluate the stability and recyclability of MOF-808@TpAzo composites in the application environment, the recycling experiments were conducted. In the six-cycle recovery experiments, MOF-808@TpAzo can still maintain an excellent photocatalytic activity for Cr(VI) reduction (Figure 4E). After the six-cycle tests, the removal capacity of MOF-808@TpAzo for Cr(VI) is still up to 98.49%. In addition, PXRD and FT-IR analyses were performed on the samples before and after six cycles (Figure S11). Although the peak intensity in the PXRD patterns of the samples after the six cycles slightly decreased as compared with that before the experiment, the characteristic diffraction peaks of MOF-808 and TpAzo can still be identified. Meanwhile, the FT-IR test can also visually show that the chemical bonds of the samples have not changed significantly before and after six cycles. The above experimental results show that, after six cycles of experiments in a strongly acidic environment, the crystal structure and chemical connections of the composite material can still maintain the structural preservation by the strongly acidic environment. After six cycles, the SEM image of MOF-808@TpAzo (Figure S12) still showed an octahedral morphology, with an irregularly shaped sheet-like structure uniformly covering the surface, showing almost no change compared to the original morphology. The concentration of Zr ions in the supernatant before and after six cycles of reactions was determined by ICP-OES (Figure S13). The results showed that the release amount of Zr in MOF-808@TpAzo only slightly increased from 0.0119 mg/L to 0.0658 mg/L. Although the release concentration increased after multiple cycles, the absolute value remained at an extremely low level (<0.1 mg/L), indicating that, even in a strongly acidic reaction medium, the MOF-808 framework could maintain good structural integrity and no significant degradation of metal nodes occurred. Combined with the unchanged FT-IR spectrum, PXRD pattern, and ICP-OES test, these results collectively confirmed that this catalyst has good stability under strong acid conditions. In addition, because the covalent connection of MOF-808@TpAzo promotes the separation and transfer of photogenerated charges, the complex shows a superior photocatalytic reduction performance of Cr(VI) as compared to the reported porous photocatalysis (Table 1). For the purpose of comparison, the reaction constants in all the prior studies have been converted to specific activity. The calculation formula can be found in the Section S2 Supplementary Formula.

In simulated wastewater, the photocatalytic reduction performance of MOF-808@TpAzo toward Cr(VI) was further investigated. Firstly, to verify the feasibility of accurately detecting the concentration of hexavalent chromium using ultraviolet–visible (UV-Vis) spectroscopy in the presence of other ions, the color development of diphenylaminourea under the conditions of single or multiple ions’ coexistence was tested. The results showed that, after the addition of one or more ions, the intensity and shape of the ultraviolet absorption characteristic peak of hexavalent chromium at 540 nm did not change significantly (Figure S14). This result confirmed that, even in the simulated wastewater with multiple ion interferences, diphenylaminourea can still be used as a UV-Vis spectrophotometric method to test the concentration of Cr(VI) in this experiment. Subsequently, the photocatalytic reduction experiment of Cr(VI) in the simulated wastewater was carried out using MOF-808@TpAzo. Figure 4F shows the ultraviolet absorption spectra of the sampled solutions at different reaction times. With the increase in time, the intensity of the absorption characteristic peak of Cr(VI) at 540 nm gradually decreases; at 120 min, the absorption peak intensity drops to nearly 0, and 98.04% of Cr(VI) in the original simulated wastewater was reduced to Cr(III) (Figure S15). Although there are multiple interfering ions in the simulated wastewater environment, MOF-808@TpAzo still exhibits an excellent photocatalytic reduction performance, demonstrating the excellent applicability and stability of MOF-808@TpAzo in simulated systems close to the composition of wastewater.

### 2.6. Mechanism of Photocatalytic Reduction of Cr(VI)

**Figure 5:**
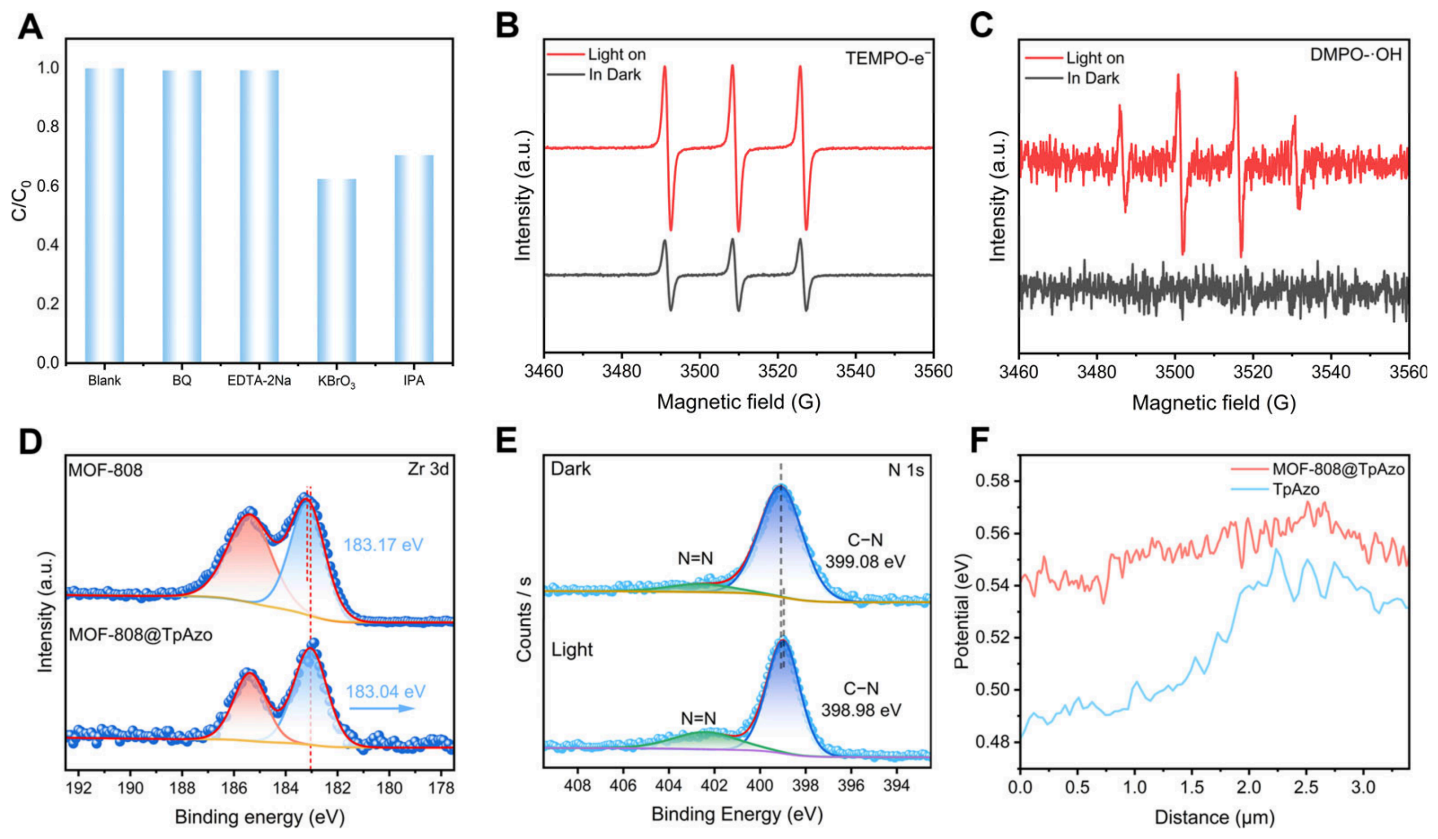
(**A**) Effects of varying free radical scavengers. (**B**) TEMPO^−^ spin trapping EPR spectra of e^−^. (**C**) DMPO^−^ spin trapping EPR spectra of ·OH. (**D**) MOF-808 and MOF-808@TpAzo Zr 3d high-resolution XPS. (**E**) N 1s in-situ XPS high-resolution deconvolution spectra of MOF-808@TpAzo. (**F**) The line scan surface potential distribution of TpAzo and MOF-808@TpAzo in Kelvin probe force microscopy (KPFM).

The possible mechanism of the MOF-808@TpAzo photocatalytic reduction of Cr(VI) was explored. By using potassium bromate (KBrO_3_), isopropanol (IPA), benzoquinone (BQ), and disodium edetate (EDTA-2Na) as capture agents, the photocatalytic reduction of Cr(VI) experiment was conducted to capture e^−^−, ·OH, ${\text{·}O}_{2}^{-}$, and h^+^ throughout the reaction process (Figure 5A). In the presence of KBrO_3_, the photocatalytic reduction efficiency of Cr(VI) by MOF-808@TpAzo is significantly inhibited, indicating that e^−^ plays an important role throughout the reaction process and participates in the direct reduction of Cr(VI). As for IPA, the photocatalytic reduction efficiency of Cr(VI) is also inhibited, but the extent of inhibition is less pronounced than that of KBrO_3_. It is speculated that ·OH indirectly participates in the reduction of Cr(VI). Meanwhile, when BQ is used as the ${\text{·}O}_{2}^{-}$ capture agent, compared with the blank group, it only slows down the initial reduction rate and does not affect the final photocatalytic reduction degree (Figure S16). ${\text{·}O}_{2}^{-}$ may significantly enhance the efficiency in the early stage of the reaction by participating in the formation of more active intermediate species or optimizing the electron transfer pathways on the catalyst surface during the reaction. This is crucial for the rapid initiation of the catalytic cycle, but, since it does not directly participate in the final reduction step of Cr(VI), it does not affect the maximum conversion rate of the reaction.

Furthermore, electron paramagnetic resonance (EPR) was used to determine the active substances produced in the photocatalytic process MOF-808@TpAzo (Figure 5B,C) [76]. Compared with the dark conditions, stronger characteristic signals of e^−^ and ·OH were detected after 15 min of illumination, proving that MOF-808@TpAzo can provide e^−^ and ·OH to participate in the reduction reaction under illumination. In addition, the signal intensity of superoxide free radicals was also detected under the light (Figure S17). It is proved that ${\text{·}O}_{2}^{-}$ was indeed generated during the reaction process. The Zr 3d_5/2_ peak slightly shifted negatively from 183.17 eV to 183.04 eV (Figure 5D). The negative shift indicates an increase in the electron cloud density of the Zr sites, that is, the Zr atoms in the composite material gained a small number of electrons and were in a more electron-rich state [77]. To elucidate the charge transfer dynamics within the MOF-808@TpAzo heterostructure, in-situ XPS was conducted. Upon irradiation, the N 1s spectra (Figure 5E) exhibit a positive shift of the C−N binding energy from 398.98 eV to 399.08 eV. This reduction in local electron density confirms that the TpAzo shell acts as an efficient electron donor, experiencing spatial electron depletion. Concurrently, the O 1s spectra (Figure S18) reveal the destination of these electrons. After irradiation, the bulk lattice oxygen (Zr−O) signal significantly attenuates, whereas the terminal Zr−OH signal remains stable. This confirms that light excites electrons to be injected into the Zr_6_ nodes of the MOF core [78]. The massive accumulation of electrons at these metal–oxo nodes induces localized structural disorder and lattice distortion [79]. This distortion, coupled with the altered charge density, modulates the coordination environment and activates the bulk lattice oxygen [80], manifesting macroscopically as the attenuation of the Zr−O peak.

To further verify the charge transfer pathway revealed by in-situ XPS, Kelvin probe force microscopy (KPFM) was used to detect the surface potential distribution of the TpAzo and MOF-808@TpAzo (Figure S19). The surface morphology and potential distribution diagrams indicated that the interface charge density of the pure lamellar TpAzo changed significantly after it was combined with MOF-808. The extracted surface potential line sweep curves (Figure 5F) visually showed that the surface potential of the MOF-808@TpAzo heterojunction was significantly higher than that of pristine TpAzo. This significant surface potential difference indicates that, at the interface formed by the contact of the two materials, electrons will spontaneously transfer from the TpAzo shell layer to the MOF-808 core with a higher surface potential. Under light excitation, this interfacial potential difference serves as a powerful driving force, promoting the photogenerated electrons to cross the core-shell interface and flow directionally from the outer layer of the TpAzo shell to the inner layer of the MOF-808 core. This clear electron migration path effectively inhibits the reverse recombination of carriers, thereby providing abundant active electrons for photocatalytic reduction in a strongly acidic environment.

**Scheme 2:**
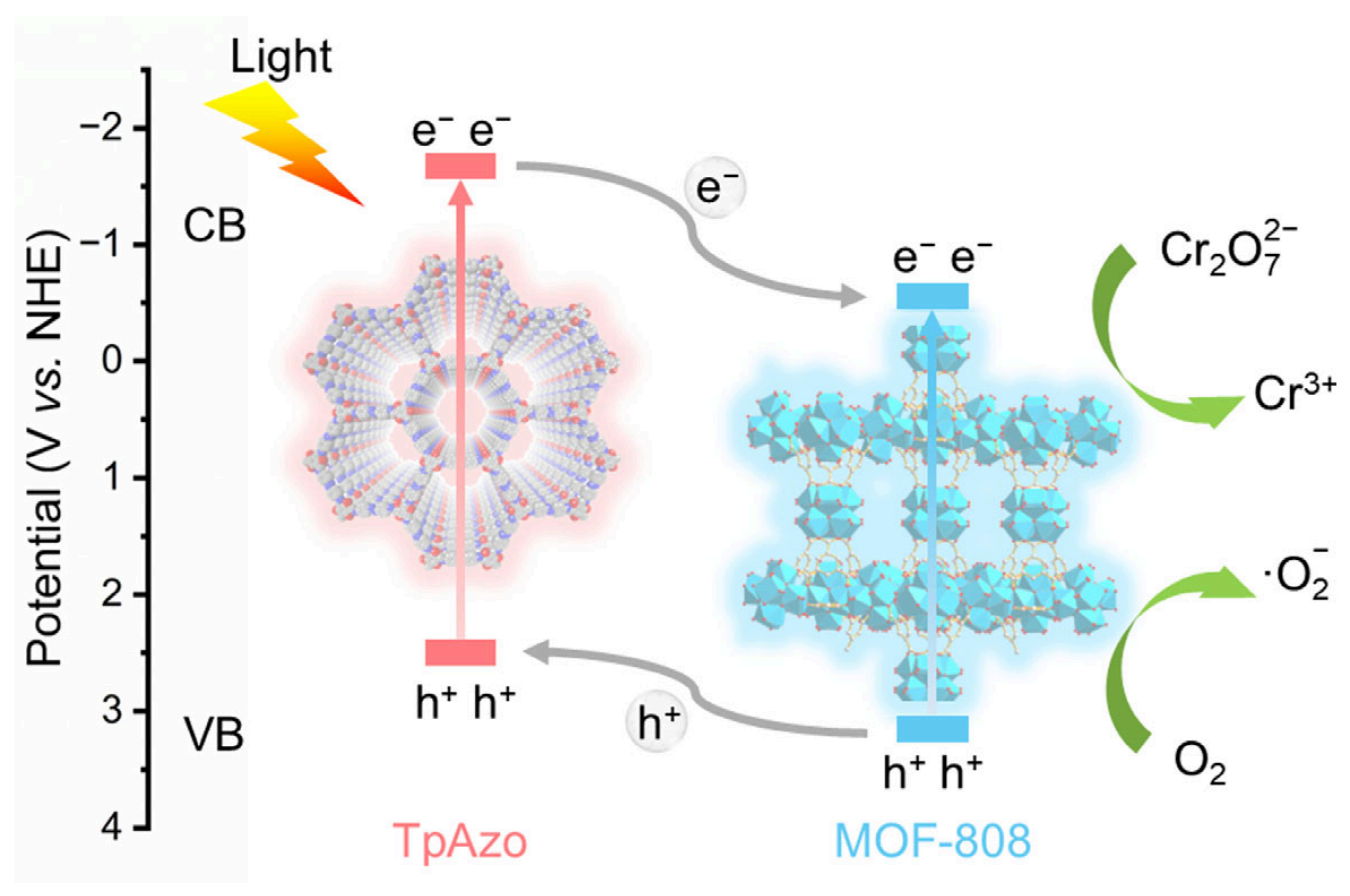
Possible mechanistic diagram of photocatalytic reduction of Cr(VI).

Based on the analysis of the results, the light catalytic mechanism can be summarized as follows. Since the MOF-808 core has almost no visible light response, the peripheral TpAzo shell mainly functions as a “light-catching antenna”. Under xenon lamp irradiation, the electrons in the valence band (VB) of TpAzo are excited to transition to its conduction band (CB). Subsequently, driven by the built-in electric field within the interface and with the help of the covalent bond bridging between the two components, these photogenerated electrons are vectorially injected into the conduction band of MOF-808. Finally, a large amount of enriched electrons are localized on the highly active Zr_6_ metal nodes, serving as a reactive site to efficiently reduce the Cr(VI) species to Cr(III). This clear spatial charge separation mechanism and the complete photocatalytic electron transfer pathway are illustrated in Scheme 2.

## 3. Materials and Methods

### 3.1. Synthesis of MOF-808

The synthesis of MOF-808 was carried out via a slightly modified procedure adapted from the literature [81]. Then, 96.6 mg ZrOCl_2_·8H_2_O and 21.0 mg 1,3,5-benzenetricarboxylic acid (H_3_BTC) were added to the round-bottomed flask. Subsequently, 10 mL of deionized water and 5 mL of trifluoroacetic acid were added, and the flask was placed in a 120 °C oil bath with a magnetic stirring bar for heating and stirring for 18 h. The obtained MOF-808 was a white powder-like crystal. It was collected by a centrifuge, washed three times with deionized water and ethanol, respectively, and then dried in an oven at 60 °C.

### 3.2. Synthesis of MOF-808-NH_2_

The amination modification procedure was adapted with minor modifications from the reported literature [56]. Then, 0.2 g of washed MOF-808 was ultrasonically dispersed in 10 mL of DMF, and 0.72 g of para-aminobenzoic acid was added; the solution reacted at 60 °C for 48 h. After the reaction was completed, it was washed several times with water and ethanol, respectively, and then dried in an oven at 60 °C for 12 h to obtain the amino-functionalized MOF-808, which was denoted as MOF-808-NH_2_.

### 3.3. Synthesis of MOF-808@TpAzo and TpAzo

The synthesis method was slightly modified based on the reported literature [82]. Different masses of MOF-808-NH_2_ (112.0 mg, 56.0 mg, 28.0 mg, and 14.0 mg), 21.0 mg 2,4,6-trihydroxy-benzene-1,3,5-tricarbaldehyde (Tp), and 35.0 mg 4,4′-azodianiline (Azo) were dispersed in 3.0 mL of binary mixed solution of mesitylene/1,4-dioxane (*v*:*v* = 1:1). The solution was ultrasonicated for 10 min to evenly disperse Tp and Azo. Then, 0.5 mL of 3 M acetic acid was added to the reaction system. Subsequently, the system was transferred to a high-temperature reactor lined with tetrafluoroethylene and heated at 120 °C for 3 days. After the temperature of the mixture dropped to room temperature, it was filtered and separated, washed several times with DMSO, water, and EtOH, and then vacuum-dried at 60 °C for 24 h. A MOF-808@TpAzo composite material was obtained in the mass ratios of MOF to COF of 2:1, 1:1, 1:2, and 1:4. The synthesis of TpAzo is similar to that of MOF-808@TpAzo composites, except that MOF-808-NH_2_ is not added during the synthesis process.

### 3.4. Photocatalytic Reduction of Cr(VI) Activity Measurement

The photocatalytic reduction of Cr(VI) method has been slightly modified based on the reported literature. The photocatalytic performances of MOF-808, TpAzo, and a series of MOF-808@TpAzo composites were evaluated at 25 ± 2 °C using a 300 W xenon lamp as the irradiation source. The photocatalytic reduction experiments were performed under direct full-spectrum irradiation using a 300 W xenon lamp without any optical filters. Then, 5 mg of the as-prepared photocatalyst was dispersed in 50 mL of 50 mg·L^−1^ K_2_Cr_2_O_7_ aqueous solution, and the initial pH of the mixture was adjusted with hydrochloric acid. The mixture was continuously stirred for 30 min in a dark environment to achieve uniform dispersion of the catalyst in the Cr(VI) solution, and then the xenon light source was turned on to irradiate the reaction system. Then, 0.1 mL of the suspension was withdrawn at regular time intervals, and the photocatalyst in the suspension was removed via filtration through a 0.22 μm filter membrane. The concentration of Cr(VI) in the filtrate was determined at 540 nm by the diphenylcarbazide method (diphenylamino urea method) using a UV-Vis spectrophotometer. Unless otherwise specified, all subsequent experiments were performed using the MOF-808@TpAzo (1:2) composite.

### 3.5. Photocatalytic Reduction of Cr(VI) in Simulated Wastewater

Apart from the tested pH = 0 and the prepared 50 mL 50 mg·L^−1^ K_2_Cr_2_O_7_ solution which contains 50 mg·L^−1^ of Zn^2+^, Ni^2+^, Na^+^, Pb^2+^, Mg^2+^, Fe^3+^, Cu^2+^, Cd^2+^, Ca^2+^, and ${\mathrm{SO}}_{4}^{2-}$, which was in accordance to the reported data [83,84,85,86], the remaining steps are the same as those in the determination of the photocatalytic reduction activity of hexavalent chromium.

## 4. Conclusions

To achieve the highly efficient reduction of Cr(VI) under strongly acidic conditions, a core-shell structure photocatalytic material MOF-808@TpAzo was developed. The azo-functional covalent organic frameworks (TpAzo) were grown on the surface of MOF-808, resulting in the heterojunction structure. The covalent bonding between the MOF-808 and TpAzo promotes interfacial electron transfer and significantly enhances the separation efficiency of photogenerated carriers. Therefore, this material exhibits superior photocatalytic performance compared to the single MOF-808 and TpAzo with a Cr(VI) removal rate of 99.42% within 120 min, which is much higher than the reported results. Furthermore, MOF-808@TpAzo maintains high catalytic activity after six cycles, demonstrating excellent stability and reusability. And, in the simulated wastewater environment with multiple ionic interferences, it still maintained a catalytic efficiency of 98.04%, demonstrating its potential for application in actual wastewater. Specifically, the photocatalytic mechanism is attributed to the formation of a Type II heterojunction, which facilitates the directional migration of photogenerated electrons from the TpAzo shell to the Zr-node active sites of the MOF−808 core, thereby facilitating efficient Cr(VI) reduction. This research provides a new strategy for the development of highly efficient photocatalysts suitable for the treatment of Cr(VI) in strongly acidic wastewater.

## Figures and Tables

**Table 1:** Photocatalytic reduction of Cr(VI) by typical MOFs and COFs.

Photocatalysts	Hole Scavenger	pH	Dosage (mg)	C_Cr(VI)_ (mg L^−1^)	V (mL)	T (min)	Reduction Efficiency (%)	k min^−1^	Specific Activity mg·g^−1^·min^−1^·L^−1^	Ref.
PCN-222(Zn)	NA	1	15	20	40	40	97.2	0.090	0.120	[69]
${\mathrm{UiO}\text{-}66\text{-}N(\mathrm{CH}3)}_{3}^{+}$	NA	2	20	10	50	30	97.9	0.099	0.049	[70]
Al-TCPP(Zn)-24 h	NA	1	15	20	40	30	99.5	0.785	1.047	[71]
JOU-6	IPA	2.3	10	9	30	100	89	0.019	0.017	[72]
JOU-7	IPA	2.3	10	9	30	100	95.2	0.031	0.028	
HDU-108	NA	3	10	10	50	120	99.9	0.040	0.040	[39]
UiO-66-NH_2_@HDU-25	NA	3	10	15	50	30	99.1	0.138	0.207	[73]
MOF-In_2_S_3_@FcDc-TAPT COFs	NA	6.8	10	15	50	30	99.8	0.431	0.646	[74]
PDI/TAPB-PDA	NA	2	10	5	50	50	97	0.239	0.120	[75]
MOF-808@TpAzo	NA	0	5	50	50	120	99.42	0.035	0.352	This work

NA: No hole scavenger was added.

## Data Availability

The original contributions presented in this study are included in the article/Supplementary Materials. Further inquiries can be directed to the corresponding author.

## Supplementary Materials

The following supporting information can be downloaded at: https://www.mdpi.com/article/10.3390/ijms27188174/s1.
