# Supplementary material for: Photocatalytic Reduction of Cr(VI) by MOF@Azo-COF Under Strongly Acidic Conditions
Source: Int J Mol Sci. 2026 Sep 14;27(18):8174. doi: 10.3390/ijms27188174 (PMC13607684; doi:10.3390/ijms27188174)
Supplement: Supplementary file 1 [file ijms-27-08174-s001.zip › ijms-4532489-supplementary.pdf]

Supporting Information

Photocatalytic Reduction of Cr(VI) by MOF@Azo-COF under Strongly Acidic Conditions

Xiaolong Gong [1,2], Guojian Ren [1,3,*],Qi Zhou [1,2], Linglong Yi [1], Qingxiang Wu [1], Yingjie Hua [4] and Qinhe Pan [1,3,5,*]

[1] Key Laboratory of Advanced Materials of Tropical Island Resources, Ministry of Education, School of Chemistry and Chemical Engineering, Hainan University, Haikou 570228, China.

[2] School of Materials Science and Engineering, Hainan University, Haikou 570228, China.

[3] State Key Laboratory of Inorganic Synthesis and Preparative Chemistry, College of Chemistry, Jilin University, Changchun 130012, China

[4] Key Laboratory of Electrochemical Energy Storage and Energy Conversion of Hainan Province, School of Chemistry and Chemical Engineering, Hainan Normal University, Haikou, 571158, China

[5] National Key Laboratory of Uranium Resources Exploration-Mining and Nuclear Remote Sensing, East China University of Technology, Nanchang 330013, China

***** Correspondence: Guojian Ren, E-mail address: rengj@hainanu.edu.cn

Qinhe Pan, E-mail address: panqh@hainanu.edu.cn

Contents

1. **Methods** 4

1.1 Reagents 4

1.2 Characterization of photocatalysts 4

2. **Supplementary Formulas** 5

2.1 Nernst calculation 5

2.2 Specific activity calculation 6

3. **Supplementary Figures** 6

Figure S1. The FT-IR spectra of p-Aminobenzoic, MOF-808 and MOF-808-NH_2. 6

Figure S2. The energy dispersive spectroscopy of MOF-808. 6

Figure S3. The energy dispersive spectroscopy of MOF-808-NH_2. 7

Figure S4. FETEM images of TpAzo. 7

Figure S5. PXRD patterns of MOF-808@TpAzo synthesized with different dosage ratios of MOF-808 and TpAzo. 7

Figure S6. Mott Schottky curves of MOF-808 and TpAzo. 8

Figure S7. High resolution deconvulated spectra of Cr 3d in MOF-808@TpAzo sample after adsorption and photocatalytic reduction. 8

Figure S8. The existence forms of Cr element in hexavalent Cr solution under different pH conditions. 8

Figure S9. $K_2Cr_2O_7$ (left) and K_2CrO_4 (right) were used as chromium sources to prepare solutions and their UV-vis spectra were obtained under different pH conditions. 9

Figure S10. The photocatalytic reduction performance and reaction rate constant of MOF-808@TpAzo under different initial Cr(VI) concentrations. 9

Figure S11. FT-IR spectra, PXRD patterns before and after six photocatalytic cycling experiments. 9

Figure S12. SEM image before and after six photocatalytic cycling experiments. 10

Figure S13. The ICP test results of Zr ions in the supernatant before and after six cycles 10

Figure S14. (Left) The absorption spectrum of Cr measured by the UV-visible spectrophotometry using diphenylaminurea. (Right) The UV absorption spectra when different types of ions are present. (The concentration of each ion is 50mg L^{-1}. The Mix curve represents the mixed solution containing Cr^{3+} and all the ions.) 10

Figure S15. The performance curve of MOF-808@TpAzo in photocatalytic

reduction of Cr(VI) in simulated wastewater. 11

Figure S16. The photocatalytic reduction of Cr(VI) performance in the presence of different free radical scavengers. 11

Figure S17. DMPO⁻ spin trapping EPR spectra of ·O2 −. 12

Figure S18. O 1s in-situ XPS high-resolution deconvolution spectra of MOF-808@TpAzo. 12

Figure S19. The Kelvin probe force microscopy(KPFM) images of TpAzo and MOF-808@TpAzo. 12

1. Methods

1.1 Reagents

All the chemicals were used without further purification. 1,3,5-benzenetricarboxylic acid, 1,5-diphenylcarbohydrazide, purchased from Shanghai Macklin. $ZrOCl_2 \cdot 8H_2O$, 2,4,6-trihydroxy-benzene-1,3,5-tricarbaldehyde (Tp), 1,3,5-trimethylbenzene, 1,4-dioxane, 4-Aminobenzoic acid were purchased from Aladdin. 4,4'-diaminoazobenzene (Azo) were purchased from Bide Pharmatech Ltd. $K_2Cr_2O_7$, N,N-dimethylformamide (DMF), Dimethyl sulfoxide (DMSO), CH_3OH, CH_3COOH was purchased from Xilong Scientific Co., Ltd. Deionized water was used throughout all experiments.

1.2 Characterization of photocatalysts

The crystal phase and purity of the material were measured by Cu Kα radiation (λ=1.54178 Å) at a temperature of 293 K by using Rigaku Supernova X-ray diffraction (XRD). The scanning speed was set at 10°/min, and the scanning range was from 2° to 50°. Infrared spectroscopy analysis of KBr wafers in the range of 4000 to 400 cm^{-1} was conducted using a Brooktens-27 infrared spectrometer. Ultraviolet-visible absorption spectroscopy (UV-vis) was determined using Shimadzu UV-2700 spectrophotometer. The morphologies of different materials were observed by using the Hitachi S3000 scanning electron microscope (SEM) and FEI Tecnai G2 F20(FETEM). The FETEM energy spectrum test uses the instrument model of Oxford XPLORE. The Kelvin probe force microscope uses the instrument model of Burker Dimension FastScan/Icon. X-ray photoelectron spectroscopy and in-situ X-ray photoelectron spectroscopy were conducted using the Thermo Fisher Escalab 250Xi X-ray photoelectron spectrometer. The ICP test uses the instrument model of Agilent ICPOES730 from the United States.

Photoelectrochemical experiments were conducted in a standard three-electrode system using the CHI 660 E (Shanghai Chenhua) electrochemical workstation as the test platform, Pt wire as the counter electrode, and saturated calomel electrode as the reference electrode. The working electrode was ultrasonically treated with 5 mg of photocatalyst powder and 1 mL of deionized water for 30 minutes, and then the resulting solution was dropped onto ITO glass for preparation. A 300W xenon lamp (PLS-SXE300, Beijing Precision Technology Co., LTD.) was used without a filter for illumination. Electrochemical impedance spectroscopy (EIS) was performed in 0.1 M KCl containing 5 mM $[Fe(CN)_6]^{3-/4-}$ within the frequency range of 0.1 Hz to 100 kHz. The photocurrent test was conducted in a 0.2 M Na_2SO_4 solution. Use a 300W xenon

lamp without a filter for illumination, and switch it every 30 seconds.

EPR test

Electron paramagnetic resonance (EPR) tests were conducted by using the Bruker A300-10/12 instrument and the capture agents (DMPO and TEMP) produced by Sigma.

Hydroxyl radicals:

A sample aqueous solution with a concentration of 1 mg/mL was prepared and ultrasonically dispersed. Then, 100 microliters of this solution were added to 100 microliters of a 100 mM DMPO solution. The mixture was thoroughly mixed, packed into a capillary tube, and sealed. Subsequently, the capillary tube was placed into a sample tube and inserted into the instrument for testing and data collection. The measurements were conducted for 15 minutes respectively in the dark and under illumination using a 300W Xenon lamp.

Photogenerated electrons:

A sample acetonitrile solution with a concentration of 1 mg/mL was prepared and ultrasonically dispersed. Following this, 100 microliters of this solution were added to 100 microliters of a 20 mM TEMPO acetonitrile solution. After thorough mixing, the sample was packed into a capillary tube, which was subsequently sealed. The capillary tube was then placed into a sample tube and inserted into the instrument for testing and data collection. The measurements were performed for 15 minutes both in the dark and under illumination using a 300W Xenon lamp.

2. Supplementary Formulas

2.1 Nernst calculation

The reduction half reaction of $HCrO_4^-$ is as follows.

$$HCrO_4^- + 7H^+ + 3e^- \rightarrow Cr^{3+} + 4H_2O$$

At a normal temperature (298.15 K), the commonly used logarithmic form of the Nernst equation is:

$$E = E^{\theta} - \frac{RT}{nF} \ln Q$$

E is the reduction potential of actual conditions. E^{θ} is the standard reduction potential. n is the number of electrons transferred in the reaction. Q is the reaction quotient.

Electron transfer number (n): According to the half-reaction, 1 molecule of $HCrO_4^-$ needs to absorb 3 electrons to be reduced to Cr^{3+}, so n = 3.

Reaction quotient (Q): $Q = \frac{\text{Reactant activity product}}{\text{Product activity product}}$, The activity of water is defined as 1.

$$Q=\frac{c(Cr^{3+})\cdot[c(H_2O)]^4}{c(HCrO_4^-)\cdot[c(H^+)]^7}$$

$$Q=\frac{c(Cr^{3+})}{c(HCrO_4^-)\cdot[c(H^+)]^7}$$

Substitute $n = 3$ and Q,

$$E=E^{\theta}-\frac{0.05916}{3}\log\frac{c(Cr^{3+})}{c(HCrO_4^-)\cdot[c(H^+)]^7}$$

2.2 Specific activity calculation

The reaction rate is $r=-\frac{dC}{dt}=kC$. At the initial moment of the reaction (t = 0), the initial concentration $C=C_0$, therefore:$r_0=kC_0$. The unit of r_0 is mmol·L^{-1}·min^{-1}

Calculate specific activity (SA)

$$SA=\frac{R_0}{N_{total}}=\frac{kC_0V}{m}$$

Unit is mg·g^{-1}·min^{-1}·L^{-1}

3. Supplementary Figures

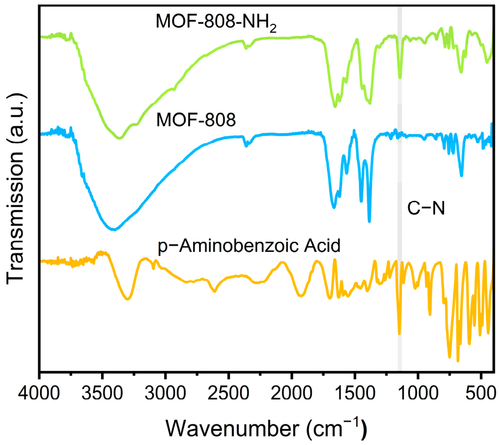

Figure S1. The FT-IR spectra of p-Aminobenzoic, MOF-808 and MOF-808-NH_2.

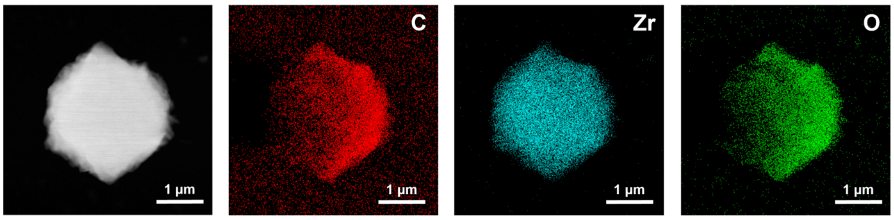

Figure S2. The energy dispersive spectroscopy of MOF-808.

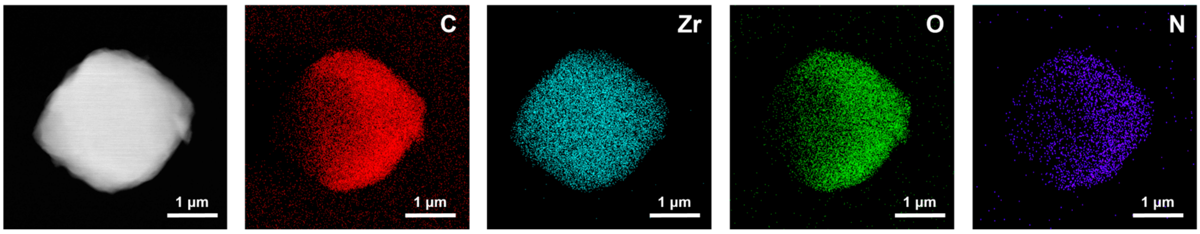

Figure S3. The energy dispersive spectroscopy of MOF-808-NH_2.

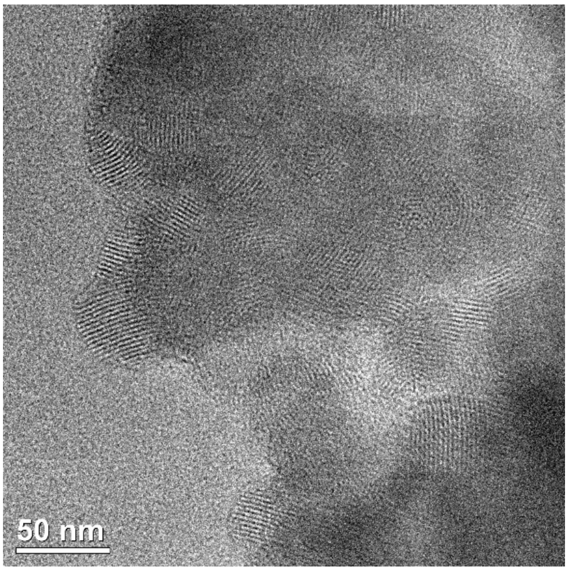

Figure S4. FETEM images of TpAzo.

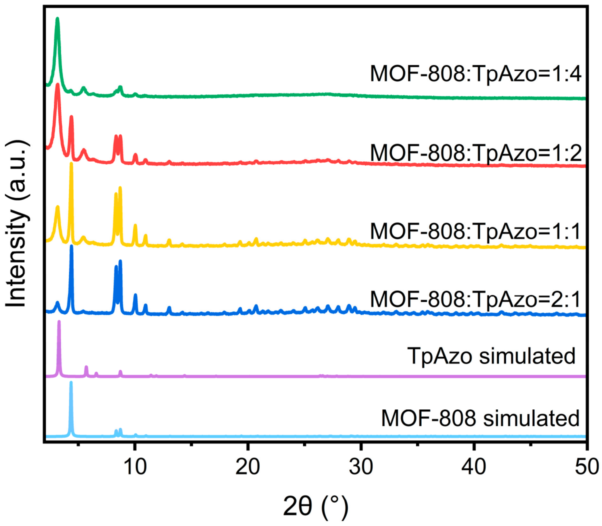

Figure S5. PXRD patterns of MOF-808@TpAzo synthesized with different dosage ratios of MOF-808 and TpAzo.

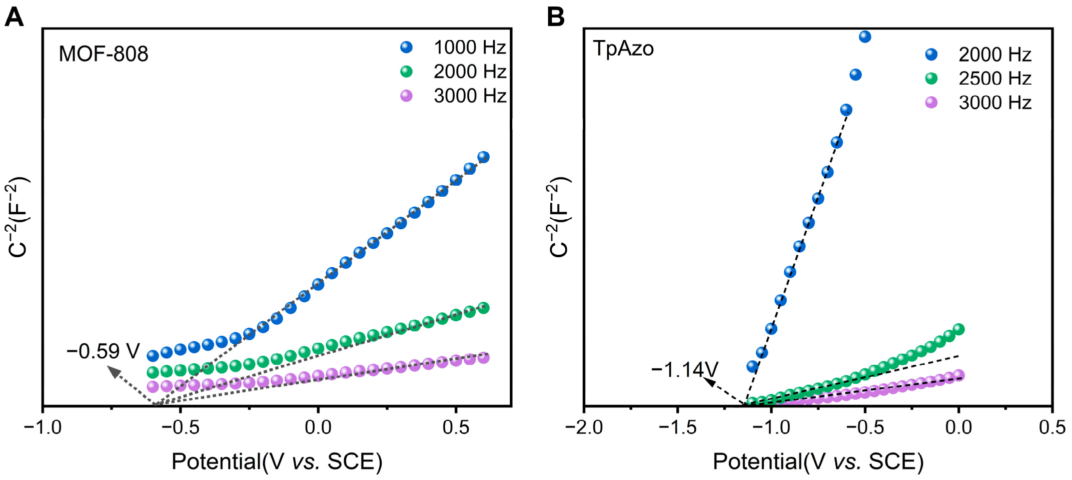

Figure S6. Mott Schottky curves of MOF-808 and TpAzo.

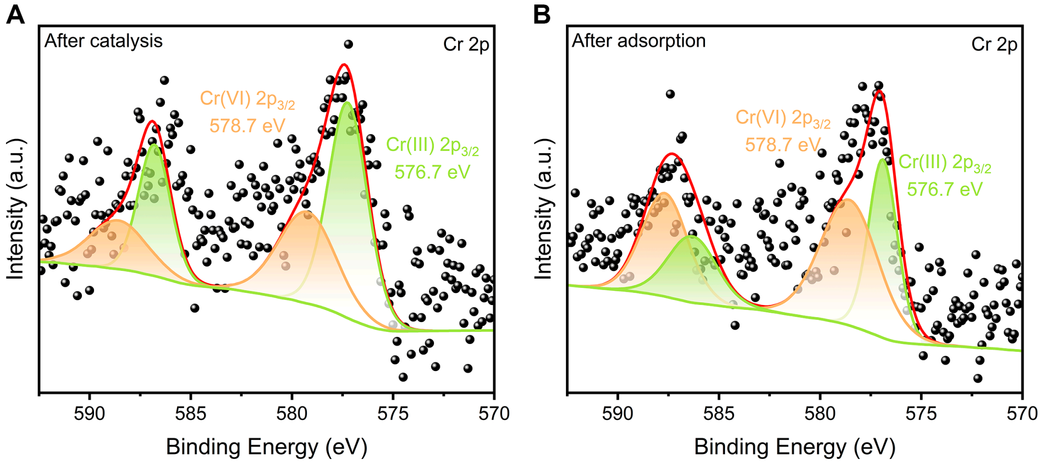

Figure S7. High resolution deconvulated spectra of Cr 3d in MOF-808@TpAzo sample after adsorption and photocatalytic reduction.

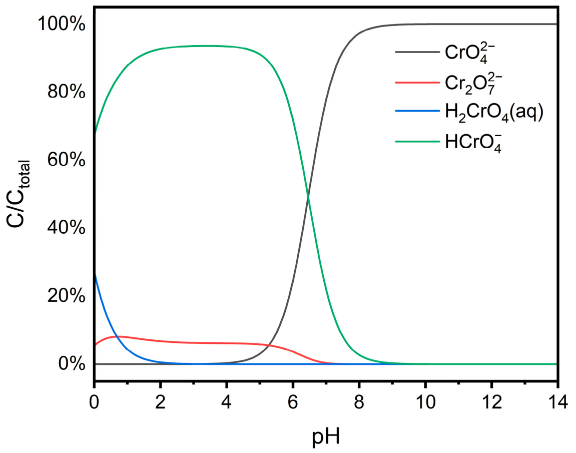

Figure S8. The existence forms of Cr element in hexavalent Cr solution under different pH conditions.

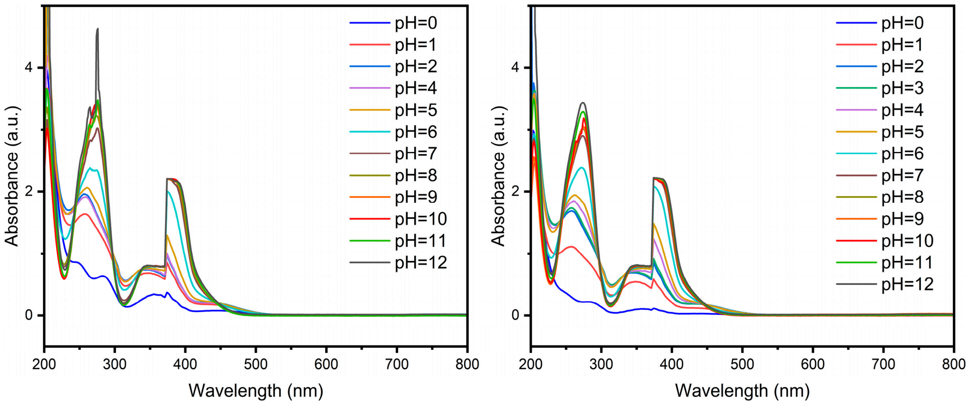

Figure S9. $K_2Cr_2O_7$ (left) and K_2CrO_4 (right) were used as chromium sources to prepare solutions and their UV-vis spectra were obtained under different pH conditions.

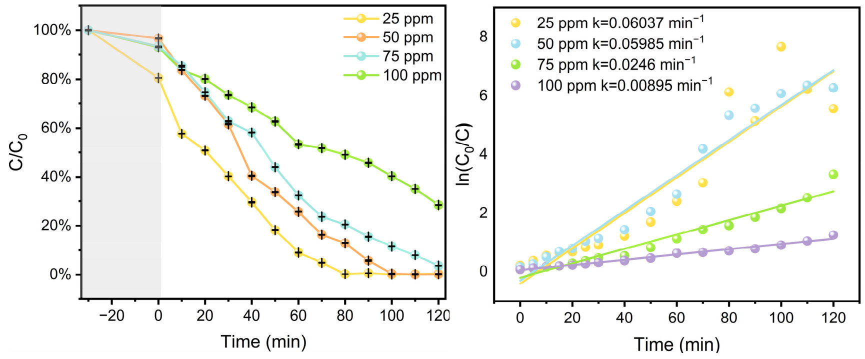

Figure S10. The photocatalytic reduction performance and reaction rate constant of MOF-808@TpAzo under different initial Cr(VI) concentrations.

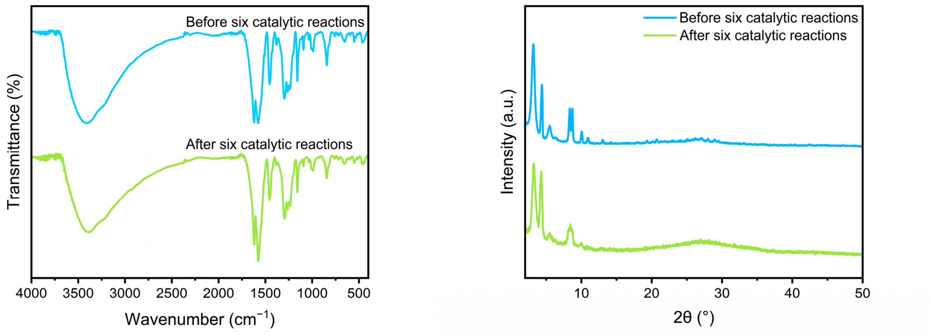

Figure S11. FT-IR spectra, PXRD patterns before and after six photocatalytic cycling experiments.

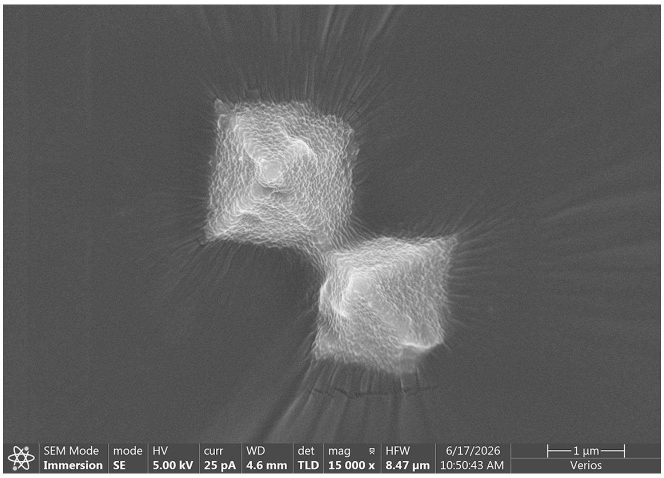

Figure S12. SEM image before and after six photocatalytic cycling experiments.

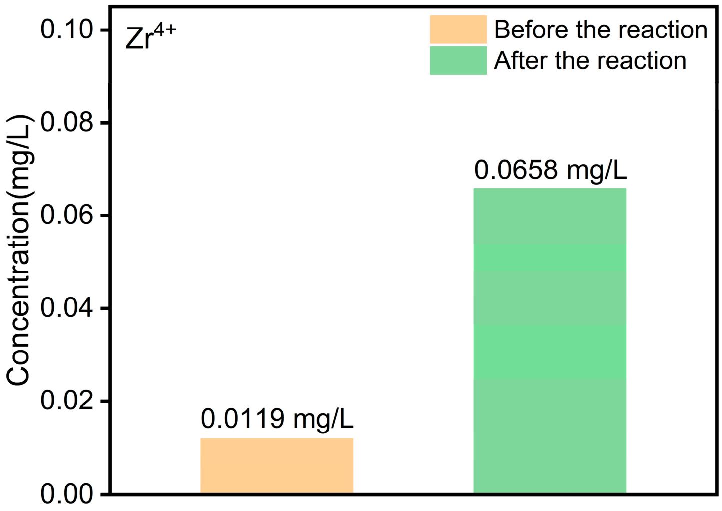

Figure S13. The ICP test results of Zr ions in the supernatant before and after six cycles

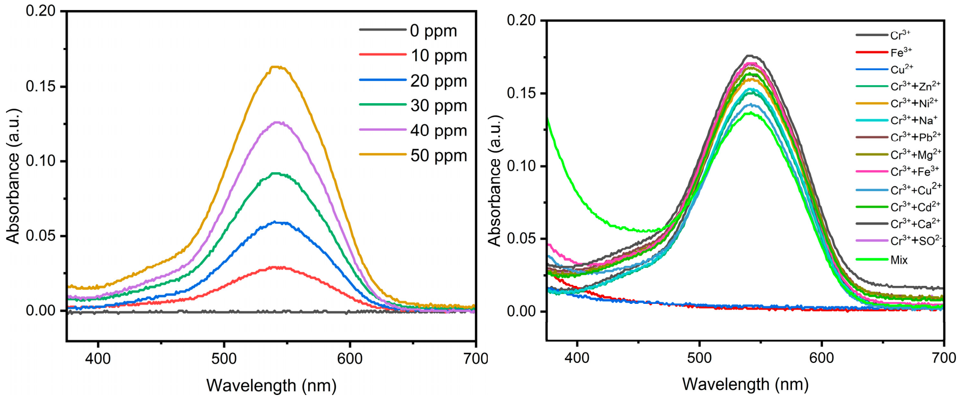

Figure S14. (Left) The absorption spectrum of Cr measured by the UV-visible spectrophotometry using diphenylaminurea. (Right) The UV absorption spectra when different types of ions are present. (The concentration of each ion is 50mg L^{-1}. The Mix curve represents the mixed solution containing Cr^{3+} and all the ions.)

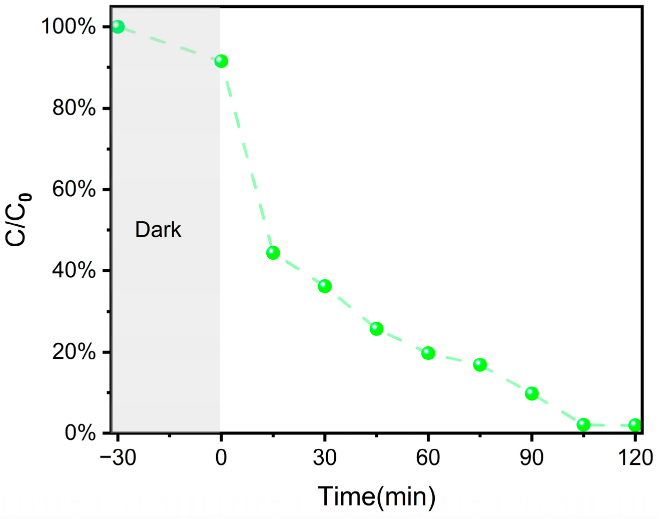

Figure S15. The performance curve of MOF-808@TpAzo in photocatalytic reduction of Cr(VI) in simulated wastewater.

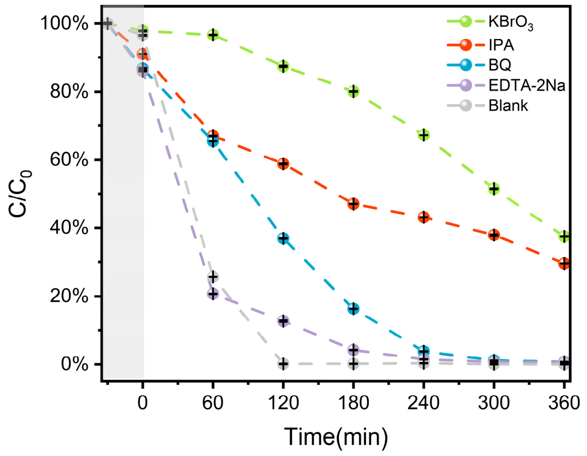

Figure S16. The photocatalytic reduction of Cr(VI) performance in the presence of different free radical scavengers.

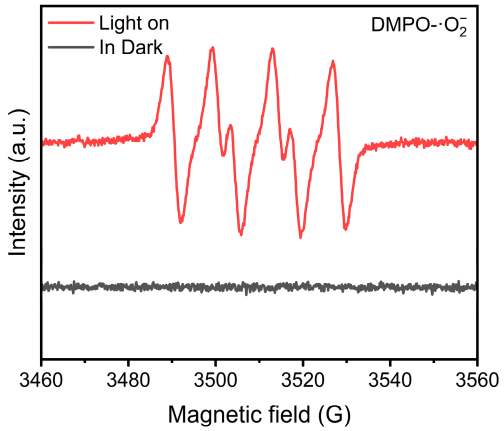

Figure S17. DMPO⁻ spin trapping EPR spectra of $·O_2^-$.

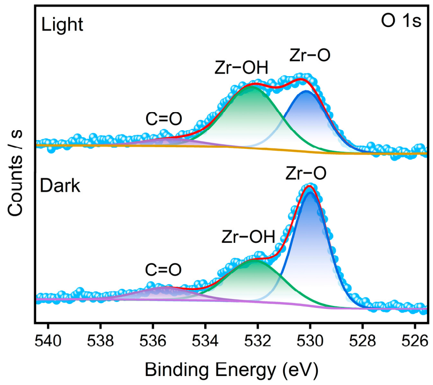

Figure S18. O 1s in-situ XPS high-resolution deconvolution spectra of MOF-808@TpAzo.

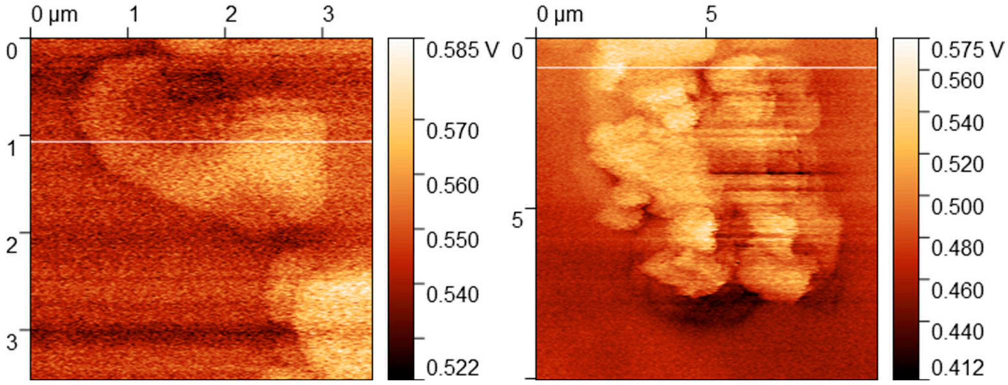

Figure S19. The Kelvin probe force microscopy(KPFM) images of TpAzo and MOF-808@TpAzo.
